# *Pseudomonas aeruginosa*, my model for research on quorum sensing, biofilms, and opportunistic infections

**DOI:** 10.1128/jb.00430-25

**Published:** 2025-12-17

**Authors:** E. Peter Greenberg

**Affiliations:** 1 Department of Microbiology, University of Washington 312771, Seattle, Washington, USA; Geisel School of Medicine at Dartmouth, Hanover, New Hampshire, USA

**Keywords:** sociomicrobiology, model organisms, cell-cell signaling

## EDITORIAL

With encouragement from the *Journal of Bacteriology* Editor in Chief, George O’Toole, I have written this short essay in a style different than *Journal of Bacteriology* articles on model organisms. It is a more personal journey about how and why *Pseudomonas aeruginosa* has been *my* model bacterium for over 30 years of research on biofilms, quorum sensing, and opportunistic infections. It can be the unwritten parts of stories that are perhaps most revealing about how a scientific field might develop. Decisions can be serendipitous and are often influenced by quite personal factors and interpersonal relationships, and this essay summarizes some of *my* unwritten parts of the story.

How the gram-negative (a gammaproteobacterium) obligate respiring bacterium *P. aeruginosa* came to be my model includes chance meetings with other microbiologists, investment from a private foundation, and a big decision I made when starting graduate school to focus on bacterial physiology rather than bacterial genetics. I was fascinated by both subdisciplines but drawn by the lure of big equipment to measure things and not by the limited genetics tools of the time, toothpicks and agar plates. It has been to my great delight and good fortune that bacterial genetics and physiology eventually dovetailed as the area of molecular biology came into existence, and I have been able to play in both sandboxes. I note that the big equipment is now in the hands of the geneticists!

To tell the story, I start with some *Pseudomonas* history. The first reports that *P. aeruginosa* was a causative agent of human infections were published in the late 1930s and early 1940s (1). *P. aeruginosa* causes a variety of infections but rarely infects uncompromised healthy humans. Rather, it can cause opportunistic infections in people with burn wounds, immunocompromised individuals, and people with other underlying conditions, like those with the genetic disease cystic fibrosis (CF). *P. aeruginosa* infections are notorious because they are very difficult to cure, and in many cases, incurable even with antibiotic therapy. The problem is vexing. Not only are some isolates resistant to antibiotics, but even when the clinical laboratory reports sensitivity, with aggressive antibiotic therapy, the infections persist. *P. aeruginosa* is one of several related species, the fluorescent pseudomonads. These species secrete a siderophore called pyoverdine, which emits a blue fluorescence when excited by UV light. In fact, for decades, blue fluorescence in a burn wound has been an informal diagnostic for poor responses to antibiotic treatment. This resistance to antibiotic treatment drew attention to *P. aeruginosa* antibiotic resistance mechanisms, and there has been intense study of inner membrane-bound efflux pumps and outer membrane porins in the species (2). This research has led to an understanding of why classical antibiotic resistance, as defined by a clinical laboratory report, is so prevalent among *P. aeruginosa* isolates.

By the 1950s and 1960s, some giants in the field of microbiology had already taken an interest in *P. aeruginosa* and the fluorescent pseudomonads. As early as 1955, the Australian bacterial geneticist Bruce Holloway (3) developed methods to study genetic recombination, transduction, and conjugation in *P. aeruginosa*. He used clinical isolates from infected patients, and descendants of some of his isolates are among the most commonly studied strains today. The general microbiologist Roger Stanier and his colleagues, Norberto Palleroni and Michael Doudoroff, at the University of California at Berkeley, became interested in the fluorescent pseudomonads for several reasons. Among these reasons, they were interested in the metabolic diversity of these bacteria. In the late 1940s and 1950s, they used fluorescent pseudomonads to study alcohol and aromatic acid oxidation (4, 5). They realized that the ability of these bacteria to metabolize many different compounds provided a handle to develop a taxonomy. At the time, bacterial taxonomy was limited. Generally speaking, bacteria were either cocci or bacilli, with some exceptions; some stained positive with Gram’s stain and others did not. There were not enough distinguishing characteristics for a taxonomist. There was, however, easily assessed phenotypic diversity among different fluorescent pseudomonad isolates, and the Berkeley team was able to group isolates into species based on their metabolic capabilities. The culmination of much of this work was a publication in the *Journal of General Microbiology* in 1966 (6). This regular article was 112 pages in length! Of course, taxonomy has changed since this publication. At the time, nucleic acid sequencing was not a reality. Phylogenetic trees were based solely on phenotypic traits. We have learned that their approach, while diagnostic, does not recapitulate phylogeny. Thus, the Berkeley team’s taxonomy grouped some disparately related genera in the fluorescent pseudomonads.

I was drawn to study the metabolic flexibility of the fluorescent pseudomonads, and this formed the basis of my Master’s degree from the University of Iowa, where I worked with George Becker on denitrification in several species of *Pseudomonas*. When at Iowa, I developed an interest in marine bacteria for reasons discussed in the next paragraph and moved to the University of Massachusetts to work toward my PhD with Ercole Canale-Parola on free-living spirochetes, which are common in the sediments of brackish waters. As a component of my PhD program, I was sent (to my pleasure) to the Marine Biological Laboratories in 1973 to take a summer course in microbial ecology. It was there I learned about cell density-dependent control of luminescence in *Vibrio fischeri* and *Vibrio harveyi* from Ken Nealson and Woody Hastings, who had just published their first paper on the phenomenon. I note that this first paper was published in the *Journal of Bacteriology* (7). I found exactly what I wanted to work on. I was fortunate that after another 3 years of finishing my PhD research, Hastings accepted me as a postdoctoral fellow in his lab at Harvard to do just that.

Why was I interested in marine microbiology, and why did I know I wanted to work on cell-density-dependent regulation of bacterial luminescence? My colleague and friend Colin Manoil encouraged me to start to answer these questions with a snippet of my life as a high school student growing up in the Seattle area. In high school, I was mainly preoccupied with my moderately successful local rock band, but a field trip to the Washington coast to explore intertidal sea life turned my head. Suffice it to say that I do not come from a family of scientists, and I had no idea that such a diversity of animal life existed on earth. I was determined to be a marine biologist. So, I quit the band to go to college and major in biology. The summer before my senior year, I enrolled in a microbiology course as an elective. I learned about the power of bacteria, not only about the bacteria that make us sick but also about the bacteria that turn the Earth’s nutrient cycles. I learned that this power was power in numbers. That these tiny little bags of enzymes could control so much of life on Earth by sheer numbers. They did not really communicate with each other or coordinate activity, or so we thought at the time. I was driven to go to graduate school and focus on microbiology, and when I knew enough, to try to learn about marine bacteria. This explains my time as a graduate student at the University of Iowa and then the University of Massachusetts. When I learned about cell density control of luminescence and how, as Hastings and Nealson explained, the coordinated control of light production required some sort of cell-cell signaling, I realized that at least these marine bacteria do communicate, and they do so to facilitate cooperation. How could I not want to work on this phenomenon?

### *P. aeruginosa*—a model for studies of quorum sensing and sociomicrobiology

I took my first faculty position in 1977 at Cornell University, where I developed a research program on the regulation of luminescence in *Vibrio fischeri*. In 1988, I moved from Cornell to the University of Iowa. This move was precipitated by the needs of our first child, who had the genetic disease CF. The University of Iowa Medical College had world-class CF care and a world-class CF research team. The clinical course of CF has changed over the decades since that move, but at the time, life expectancy for a CF patient was in the teens, and the disease was characterized by chronic *P. aeruginosa* lung infections that ultimately led to respiratory failure. My research group was focused on cell-density-dependent expression of genes required for light production in the luminescent marine bacterium *Vibrio fischeri*. By this time, Engebrecht et al. (8) had cloned the *V. fischeri* luminescence genes and discovered two genes required for cell-density-dependent regulation of luminescence, *luxR* and *luxI*. These genes were adjacent to each other. This led to my teams great success in discovering all about how the *luxR* and *luxI* gene products worked to make and respond to the chemical signal and activate the luminescence genes.

I was back at Iowa, where I had worked with fluorescent pseudomonads as a graduate student, but I had no intention to work with *P. aeruginosa*. By the early 1990s, I was helping in an administrative capacity at the University of Iowa CF Research Center. I learned from my colleague Charles Cox, a *P. aeruginosa* researcher, who was on a sabbatical working at the University of Rochester with Barbara Iglewski, an authority on *P. aeruginosa* pathogenesis and toxins, that the Iglewski group had discovered a *P. aeruginosa* transcription factor that activated a battery of virulence genes. The amino acid sequence of that activator was related to LuxR. Because it regulated the gene coding for elastase, *lasB*, it was called LasR (9). This finding led to a fruitful collaboration between the Iglewski and Greenberg teams. At that time, in the early 1990s, DNA sequencing was nothing like it is today. It involved the use of substantial quantities of radioactive phosphate and long polyacrylamide gels for Sanger sequencing. Nothing was automated. Cloning and sequencing of DNA adjacent to *lasR* led to the discovery of a *luxI* homolog that was called *lasI* (10). The collaboration showed that, like LuxI, LasI catalyzed the synthesis of an acyl-homoserine lactone quorum-sensing signal. The Lux signal was *N*-3-oxohexanoyl-homoserine lactone (11), and the Las signal was *N*-3-oxododecanoyl-homoserine lactone; it had a longer acyl side chain than the *V. fischeri* chemical (12). At that time, other investigators were beginning to discover related systems in other bacteria, and our *Journal of Bacteriology* minireview introduced the use of the term quorum sensing and response to refer to the growing group of LuxR-LuxI-type systems (13). The fact that the *P. aeruginosa* system controlled virulence gene expression in a human pathogen pushed this bacterium to the forefront as a model for studies of quorum sensing.

### *P. aeruginosa*—a model for molecular genetic studies of biofilms

A chance meeting between William Costerton and me triggered a collaboration that helped center attention on *P. aeruginosa* as a model for biofilm research. Costerton was focused on biofilms, and I on quorum sensing; both bacterial behaviors had an element of sociality. We first met at an *Annual Reviews of Microbiology* Editorial Board meeting in Palo Alto in 1989 and learned that we each had children with CF, and that we both were thinking about sociality in groups of bacteria. Later, after my group learned about quorum sensing in *P. aeruginosa*, we decided to team up to ask whether quorum sensing influenced biofilm development. Costerton had proposed that CF lung infections were impossible to cure because they represented biofilm infections, and so we used what had already become my model for quorum-sensing studies, *P. aeruginosa*. The collaboration was undertaken by Matthew Parsek, then a postdoctoral in my laboratory, and David Davies, then a postdoctoral in the Costerton group. Parsek traveled from the University of Iowa to Montana State University with *P. aeruginosa* quorum-sensing mutants carrying a plasmid he constructed to constitutively express *gfp*. Together, Davies and Parsek used the Montana State confocal imaging facility to show that *P. aeruginosa* quorum-sensing mutants formed abnormal biofilms. Our publication in *Science* (14) was notable in that it introduced the idea of using molecular genetics to understand the biology of biofilm maturation, and it was the first report in which GFP fluorescence was used for improved imaging of living bacterial biofilms. At that time, O’Toole and Kolter (15–17) were taking somewhat similar approaches to study the plant-associated bacterium *Pseudomonas fluorescens* and *P. aeruginosa*. O’Toole began to focus specifically on *P. aeruginosa*. His decision was surely based on the foundational genetic work of Holloway and the rapid advances in the 1990s on the use of modern genetics tools to study *P. aeruginosa* (18, 19).

### The CF Foundation makes a major commitment

In the late 1990s, while *P. aeruginosa* was becoming a model for studies of quorum sensing and biofilm development, a group of investigators focused on CF lung infections received a substantial financial commitment from the CF Foundation to generate a complete DNA sequence of the *P. aeruginosa* strain PAO1 genome. The genome sequence was finished and published in 2000 by a team of Seattle scientists led by University of Washington geneticist Maynard Olson (20). Although this accomplishment was just 25 years ago, the intervening period has seen a revolution in DNA sequencing technology. For reasons not related to this article, the CF Foundation has been transformed from operating on a shoestring budget to an organization with a major endowment. In the late 1990s, the Foundation and the scientific team took a big risk—a monetary risk for the Foundation and a scientific risk for the science team. At the time of publication, the PAO1 genome sequence was the largest ever completed. In the past 20 years, the cost of completing a bacterial genome has been reduced by 4–5 orders of magnitude, from millions of dollars to less than 100 dollars, and we now have available genomes of hundreds of different *P. aeruginosa* isolates, but it was this singular publication that sparked innovation and research in the ensuing years. Annotation of the sequence showed a great abundance of genes coding for a variety of antibiotic efflux pumps. There were also more genes coding for transcription regulators than in the few other bacterial genomes sequenced at that time. Now there was information to advise researchers on how to investigate mechanisms of antibiotic resistance, and there was emphasis on the metabolic plasticity implied by the multitude of genetic regulators. In 2005, I returned to my hometown of Seattle, drawn to the University of Washington and its incredible strengths in *P. aeruginosa* biology, CF research, and genomics. I remain here today as the Nester Professor of Microbiology.

Encouraged by the success of the genome sequencing project, the CF Foundation supported the development of genome-based tools. These included the creation of ordered transposon mutant libraries (21, 22) and the development of a commercial chip for transcriptomic studies. Not only did the Foundation support production of these chips, but they underwrote much of the cost of purchasing the chips for investigators *if* those investigators agreed to deposit their results in an accessible repository. Reiterating a theme, now times have changed, transcriptomics technology has seen major advances, costs have come down, and there are public data repositories designed to house results as required by reputable journals. The Foundation led the way. The Foundation’s investment was transformational for me and for the quorum-sensing field. We were able to identify genes activated by quorum sensing, determine the elements involved, and much more. We were able to determine the specificity of quorum-sensing inhibitors and understand aspects of the evolution of the quorum-sensing regulon.

From the time I first learned about luminescent marine bacteria, I have been fascinated by the idea that bacteria can communicate, and this allows groups to coordinate cooperative activities. It seemed apparent that this was the case for luminescent bacteria. The energy spent on light production by bacteria in low population density environments would not be useful. It is not visible to other creatures. As *P. aeruginosa* became a model for studies of quorum sensing, we learned that it depended on quorum sensing to activate the production of a battery of extracellular factors, exoproteases, toxic compounds like cyanide, and antimicrobial molecules, to name some factors. This has enabled research showing that indeed *P. aeruginosa* quorum sensing serves to coordinate cooperation. *P. aeruginosa* has become a model for studies revolving around the molecular basis for cooperative activities and the constraints on evolution and stability of cooperation (23).

Of course, my group has worked with many other bacteria so that we could better understand the diversity of acylhomoserine lactone signals, which exist in nature, and the roles these signals play in the fitness of bacteria. It is the fundamental knowledge we gained by putting a focus on *P. aeruginosa* that enabled much of our other successes. To finish, I need to remind the reader that this short story of *P. aeruginosa* as *my* model organism certainly does not cover the breadth of research on this bacterium and all of the reasons many microbiologists use it as *their* model organism. Rather, I hope I have provided a glimmer of insight about how seemingly disparate things in science and outside of science can lead to innovation and discovery, and a glimpse into the serendipity surrounding the discovery process.
